# Assessing haemorrhage-critical values of cerebral blood flow by modelling biomechanical stresses on capillaries in the immature brain

**DOI:** 10.1038/s41598-020-71087-7

**Published:** 2020-08-26

**Authors:** Irina Sidorenko, Varvara Turova, Nikolai Botkin, Andrey Kovtanyuk, Laura Eckardt, Ana Alves-Pinto, Ursula Felderhoff-Müser, Esther Rieger-Fackeldey, Renée Lampe

**Affiliations:** 1 Mathematical Faculty, Chair of Mathematical Modelling, Technical University of Munich, Boltzmannstr. 3, 85748 Garching, Germany; 2 School of Medicine, Klinikum Rechts Der Isar, Orthopedic Department, Research Unit for Pediatric Neuroorthopedics and Cerebral Palsy of the Buhl-Strohmaier Foundation, Technical University of Munich, Ismaninger Str. 22, 81675 Munich, Germany; 3 Department of Pediatrics I, Neonatology, Pediatric Intensive Care, Pediatric Neurology, Department of Pediatrics III, Pediatric Oncology, University Duisburg-Essen, University Hospital Essen, Hufelandstraße 55, 45147 Essen, Germany; 4 School of Medicine, Klinikum Rechts Der Isar, Department of Pediatrics, Technical University of Munich, Ismaninger Str. 22, 81675 Munich, Germany

**Keywords:** Paediatric research, Preterm birth

## Abstract

The development of intraventricular haemorrhages (*IVH*) in preterm newborns is triggered by a disruption of the vessels responsible for cerebral microcirculation. Analysis of the stresses exerted on vessel walls enables the identification of the critical values of cerebral blood flow (*CBF*) associated with the development of *IVH* in preterm infants. The purpose of the present study is the estimation of these critical *CBF* values using the biomechanical stresses obtained by the finite element modelling of immature brain capillaries. The properties of the endothelial cells and basement membranes employed were selected on the basis of published nanoindentation measurements using atomic force microscopes. The forces acting on individual capillaries were derived with a mathematical model that accounts for the peculiarities of microvascularity in the immature brain. Calculations were based on clinical measurements obtained from 254 preterm infants with the gestational age ranging from 23 to 30 weeks, with and without diagnosis of *IVH*. No distinction between the affected and control groups with the gestational age of 23 to 26 weeks was possible. For infants with the gestational age of 27 to 30 weeks, the *CBF* value of 17.03 ml/100 g/min was determined as the critical upper value, above which the likelihood of *IVH* increases.

## Introduction

One of the most frequent complications during the postnatal development of preterm infants is intraventricular haemorrhage (*IVH*). This is one of the possible causes of cerebral palsy, a medical condition characterized by lifelong motor function disorders and constituting the main source of disabilities during childhood^1^. There is evidence^2,3^ of the origin of *IVH* occurring in a highly vascularized region of the subependymal part of the brain called the germinal matrix (*GM*). The *GM* plays an important role in fetal brain development, increasing in volume up to 5% of the entire brain volume until the 23th week of gestation. The *GM* reduces rapidly after the 28th week of gestation (*WG*), disappearing fully by the 34th *WG*^4^. Several experimental studies have shown that capillaries in the *GM* present structural features that differentiate them from the other brain capillaries (non-*GM* capillaries). For example, electron-microscope observations have shown that the density of micro vessels in the *GM* is about 1.5 times higher than that of any other part of the brain and that the average diameter of vessels is also larger than in the cortex^5–7^. These and other features convey specific mechanical properties to the *GM* capillaries and, with the dynamic characteristics of blood flow in these capillaries, may underlie the higher likelihood of cerebral haemorrhages in preterm infants. The present study addresses this possibility by assessing, via mathematical modelling^8,9^, the biomechanical forces exerted on the walls of capillaries in the immature brain. In particular, the finite element method (*FEM*) was employed to describe and analyse the extent to which the structure and composition of capillaries in the *GM*, as well as the biomechanical stresses to which they are subjected, determine the likelihood of *IVH*. The assembly of a finite element model is based on several geometrical and material characteristics. In the present work, we model brain capillaries. Many studies^5,10–13^ have proved that the characteristics of the capillary network and development of *IVH* are similar across mammals, which supports using measurements from the animal studies^5,14–16^ to describe the geometry and material properties of brain capillaries.

Capillaries are the smallest blood vessels which connect arterioles and venules. They are essentially extensions of the inner linings of these larger vessels. Brain capillaries have a number of important differences to capillaries in other organs^17^. Unlike in systemic capillaries, the endothelial cells in brain capillaries are joined together by tight junctions and form a continuous layer that effectively separates the plasma from the interstitial fluid of the brain (blood–brain barrier). The innermost layer of the capillary walls, called the endothelium, is formed by single-layered cells, which cover the whole interior surface that comes into direct contact with the blood^18,19^. When subjected to the flowing of blood, endothelial cells show a marked elongation and orientation in the direction of flow. As a result, their resistance to external forces such as blood pressure and shear stress increases^14,20,21^. The endothelial cells are surrounded by a tough basement membrane, which consists essentially of an irregularly arranged felt-like meshwork of fibres and attaches the capillary to the surrounding tissues^15,16^. The basement membrane prevents capillary rupture during osmotic or hydrostatic stress, and with its rupture, a haemorrhage occurs.

In the present study, the biomechanical characteristics of endothelial cells and of the basement membrane were taken from the literature describing different nanoindentation experiments using an atomic force microscope (*AFM*)^14–16,20–22^. The biomechanical forces acting on vessel walls result from both hydrostatic pressures and shear stresses. The former exerts a force that is orthogonal to the vessel wall and imposes a circumferential stress (hoop stress) on the vessel, whereas the latter arises from the frictional drag and shear stress from flowing blood, being parallel to the endothelium in the direction of blood flow. The biomechanical forces acting on individual capillaries were estimated using a mathematical model of the cerebral blood flow (*CBF*)^8,9,23^. The model is based on records of mean arterial pressure (*MAP*) and partial pressure of carbon dioxide (*pCO*_2_) collected during the clinical monitoring of 254 preterm infants with the gestational age ranging from 23 to 30 *WG*. A receiver operating characteristic (*ROC*) analysis of stress values calculated from two groups (infants with and those without *IVH*) was then employed to separate the groups, and in this way to derive the critical values of *CBF* associated with the development of *IVH*.

## Results

### Hydrostatic blood pressure, shear stress and capillary diameter as a function of *WG*

**Figure 1:**
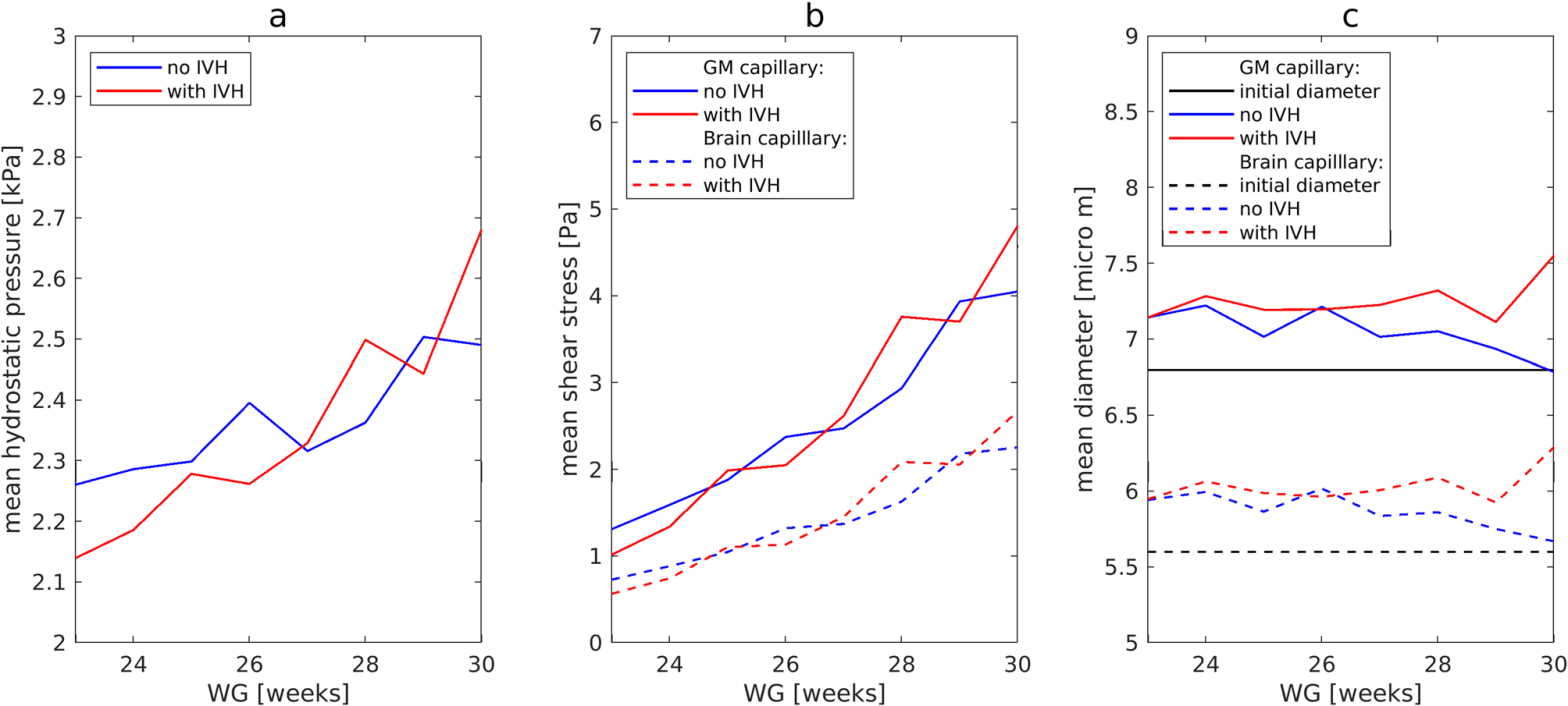
Computed values of mean hydrostatic pressure (**a**), shear stress (**b**), and capillary diameter (**c**) in brain capillaries including the *GM* capillaries.

Figure 1 presents the mathematically calculated values of hydrostatic blood pressure $P$, the shear stress $\tau$ and the diameter $d$ for capillaries in the *GM* (solid lines) and in the non-*GM* (dashed lines), averaged across infants for each *WG*, for the control (blue curves) and affected (red curves) groups. The hydrostatic blood pressure was equal for all capillaries and increased with gestational age (Fig. 1a). For the infants from the control group with *WG* = 30 this pressure reached the value of $P$ = 2.5 kPa (19 mmHg), which is close to the mean value of blood colloid osmotic pressure of 23 mmHg measured for healthy infants one month old^24^. The shear stress (Fig. 1b) demonstrated a similar dependence on *WG*, apart from an offset between capillaries of the *GM* and other brain capillaries. The mean shear stress (Fig. 1b) in capillaries of the *GM* increased with gestational age from 1.5 to 4 Pa.

**Figure 2:**
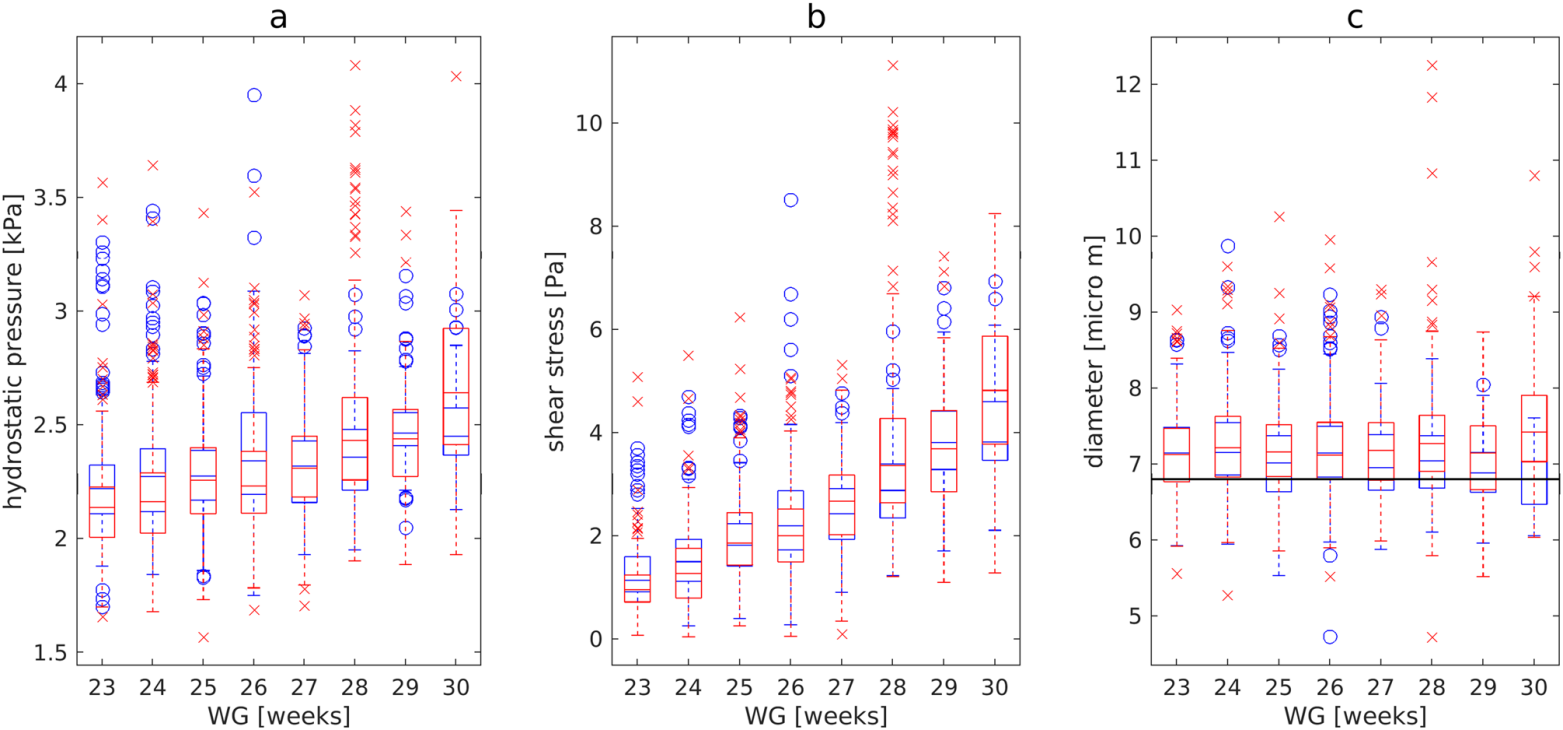
Box plots showing the hydrostatic pressure (**a**), shear stress (**b**), and diameter (**c**) for a *GM* capillary for the control (blue circles) and affected (red crosses) groups.

The reaction of the vessels to changes in *MAP* and *pCO*_2_ is seen by comparing the mean diameter (Fig. 1c), calculated with the mathematical model (red and blue curves), with the initial diameter (black lines). The larger diameter values indicated that capillaries were dilated for all *WG*. However, in the control group (blue curves) the mean diameter decreased with increasing of gestational age beyond 26 *WG* reaching the initial value ${d}_{0}$ by 30 *WG*, while in the affected group the mean diameter remained larger for all *WG*. No statistically significant differences (Wilcoxon rank-sum test) in mean hydrostatic pressure and shear stress were detected between the control and affected groups, but mean diameter was significantly higher in the affected group (control: ${d}^{GM}$ = 7.05 ± 0.15 μm, affected: ${d}^{GM}$ = 7.25 ± 0.14 μm, p = 0.021). One can see from the box plots for *GM* capillaries (Fig. 2) that almost all outlets lie above the upper whiskers for all three parameters calculated, both in the control and affected groups.

### Von Mises stress and *CBF* critical values

**Figure 3:**
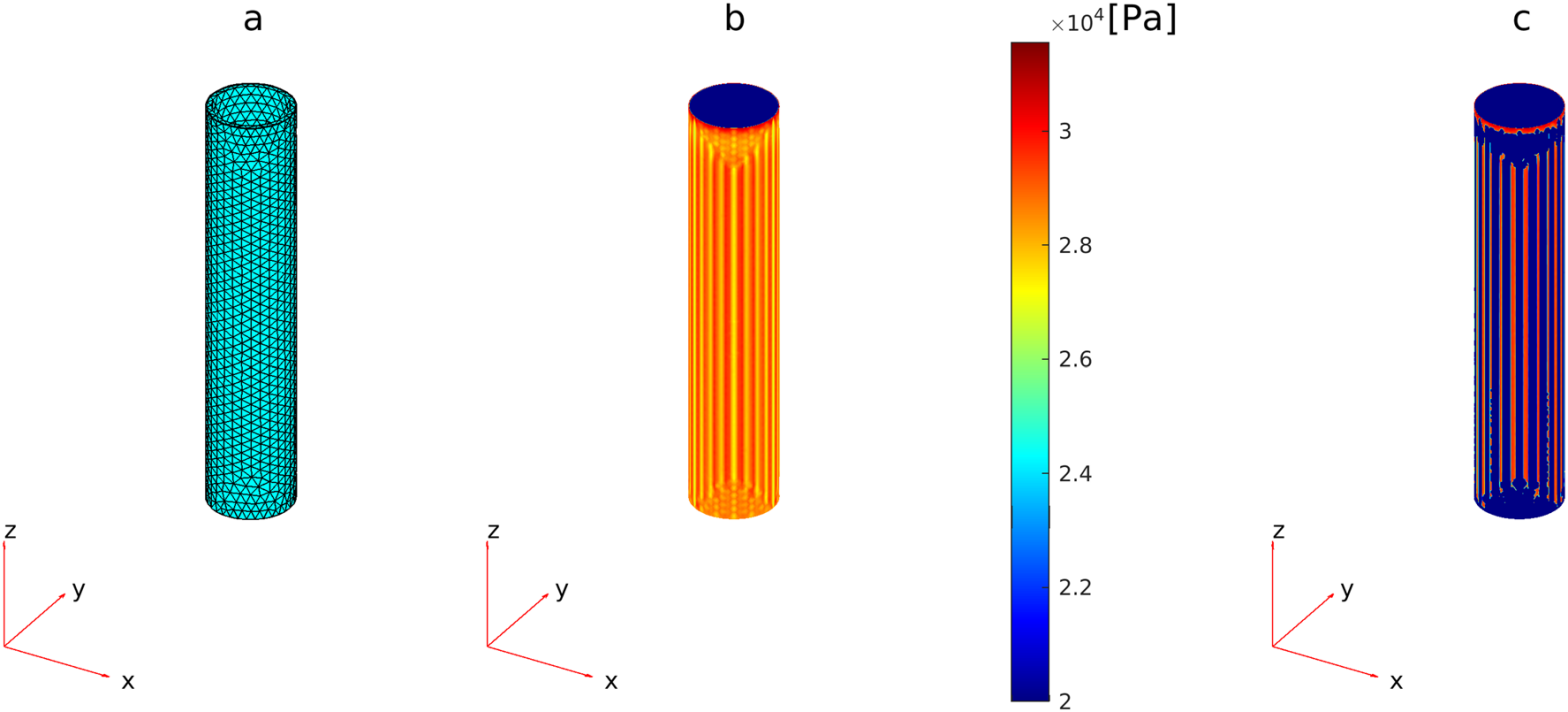
Finite Element (*FE*) simulation of the *GM* capillary. (**a**) *FE* model (geometry, node location and mesh elements) of a *GM* capillary. (**b**) The distribution of von Mises stresses ${S}_{vM}$ along the capillary wall of a *GM* vessel obtained from *FEM* calculations with fixed capillary ends for exemplary values ${P}_{EXT}$ = 0.8 kPa^25,26^, $P$ = 2.25 kPa, $\tau$ = 1.5 Pa, and diameter $d$= 7.1 μm corresponding to mean values of pressure, shear stress and capillary diameter of the control group with gestational age 23 weeks (Fig. 1). (**c**) Location of nodes (10% from mesh) with ${S}_{vM}$ > 94% $max\left({S}_{vM}\right)$. (All plots were generated with the MATLAB R2019a standard function pdeplot3D).

Figure 3b shows the distribution of von Mises stresses in the wall of a *GM* capillary obtained from *FEM* calculations with fixed capillary ends for exemplary values ${P}_{EXT}$ = 0.8 kPa^25,26^, $P$ = 2.25 kPa, $\tau$ = 1.5 Pa, and $d$ = 7.1 μm, corresponding to mean values of pressure, shear stress, and capillary diameter in the control group with the gestational age of 23 weeks (see Fig. 1). Maximum values were concentrated in the external basement membrane (Fig. 3b). The 10% of nodes with ${S}_{vM}$ > 94% $max\left({S}_{vM}\right)$ are furthermore shown in Fig. 3c.

**Figure 4:**
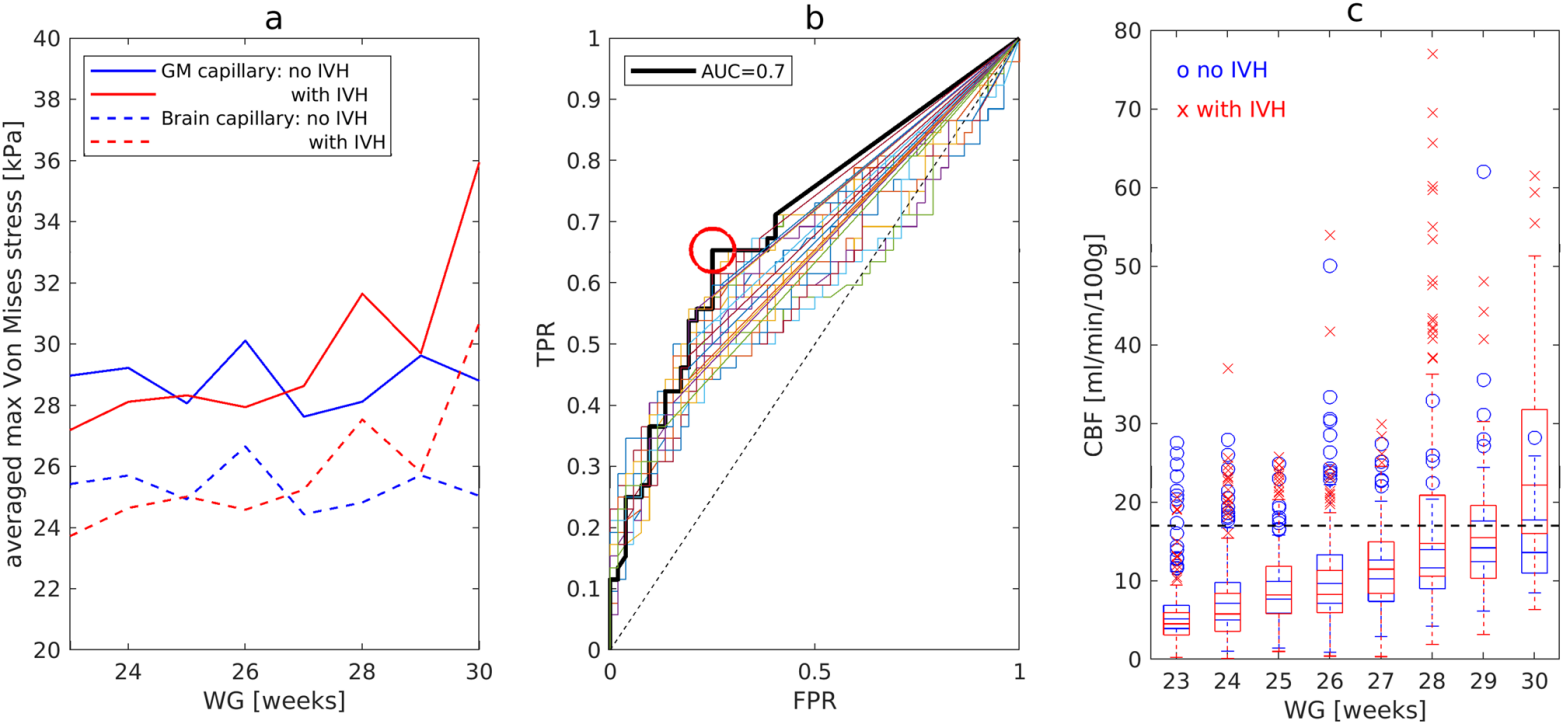
Statistical results. (**a**) Average maximum von Mises stress in brain capillaries. (**b**) *ROC* curves for ${S}_{U}$ values in range 30–40 kPa for infants from 27 to 30 *WG*. The bold curve shows the best result with *AUC* = 0.7 for ${S}_{U}$ = 33.5 kPa. The optimal point at threshold ${N}_{th}^{F}$ = 2.98% with *TPR* = 0.65 and *FPR* = 0.25 is marked with a red circle. (**c**) Box plot for computed *CBF*. Critical *CBF* = 17.03 ml/100 g/min is shown as black dashed line.

Importantly, the maximum von Mises stress (Fig. 4a) in *GM* capillaries was significantly higher than that in non-*GM* capillaries (Wilcoxon rank-sum test; *GM*, all patients: ${S}_{VM}$ = 28.96 ± 5.89 kPa; non-*GM*, all patients: ${S}_{VM}$ = 25.43 ± 4.99 kPa, p < 0.001). One can see from Fig. 4a that the stresses in *GM* capillaries and non-*GM* capillaries showed parallel behaviour with some offset. Either of the capillary types could be used for the distinction between the control and affected groups. However, the capillaries with larger stresses (*GM* capillaries in our case) should be used for the estimation of the critical *CBF* values.

The Wilcoxon rank-sum test of the maximum von Mises stress in *GM* capillaries (Fig. 4a) showed that the difference between the control and affected groups was not significant (*GM*, control: 28.87 ± 5.07 kPa; *GM*, affected: 29.04 ± 6.45 kPa, p = 0.1). Thus, the maximum von Mises stress alone was not sufficient to predict occurrence of *IVH* and, therefore, a *ROC* analysis with two variables, ultimate stress ${S}_{U}$ and percentage of failed nodes ${N}^{F}\left({S}_{U}\right)$, was conducted. In order to differentiate between the control and affected groups, the percentage of failed nodes ${N}^{F}$ as a function of the ultimate stress ${S}_{U}$ was calculated and analysed with the *ROC* method for ${S}_{U}$ in the range from 30 to 40 kPa (Fig. 4b). Given that mathematical calculations had shown that the average blood velocity in the affected group of infants with the gestational age of 23 to 26 *WG* was 2.3 times lower than that for infants with the gestational age of 27 to 30 *WG* (46.79 ± 26.40 μm/s and 107.29 ± 51.90 μm/s respectively), the *ROC* analysis was performed separately for infants with the gestational age of 23 to 26 *WG* and that of 27 to 30 *WG*.

No discrimination between the affected and control groups for infants with the gestational age of 23 to 26 *WG* was possible, for any of the values tested. For infants with the gestation age of 27 to 30 *WG*, the maximum area under the curve *AUC* = 0.7 was achieved for ${S}_{U}$ = 33.5 kPa, which is approximately one standard deviation from the maximum von Mises stress averaged across the control group (*GM* control 27–30 *WG*: ${S}_{VM}$ = 28.29 + 4.30 kPa). The optimal values of the true positive rate *TPR* = 0.65 and of the false positive rate *FPR* = 0.25 were observed for the threshold ${N}_{th}^{F}$ = 2.98%. Thus, the minimum value of *CBF* = 17.03 ml/100 g/min, at which 2.98% of mesh nodes had ${S}_{VM}\ge 33.5$ kPa, was defined as the critical upper value of *CBF* above which *IVH* may occur. One can see in the box plot of *CBF* (Fig. 4c) that in the affected group, starting from the gestational age 24 *WG*, all upper whiskers and outlets lie above the critical value of *CBF* = 17.03 ml/100 g/min obtained.

### The effect of model assumptions

The effect of the fixed and free boundary conditions at the capillary top end as well as the effect of varying ${Y}_{M}$ is presented in Table 1. Despite the changes in ultimate stress ${S}_{U}$, the *ROC*-based discrimination yielded only a 5.7% change in *CBF* critical value.

**Table 1:** Effects of varying boundary conditions and of *Y*_*M*_ on *CBF* critical value.

Boundary condition on capillary end	${Y}_{M}$(kPa)	${S}_{U}$(kPa)	*AUC*	${N}_{th}^{F}$	TRP	FRP	*CBF* (ml/100 g/min)
Fixed	100	33.5	0.70	2.9	0.65	0.25	17.30
Free	100	39	0.68	2.3	0.56	0.19	18.28
Fixed	150	40	0.69	10	0.63	0.25	17.30
Fixed	50	25.5	0.64	1.4	0.42	0.15	18.28

## Discussion

The present study estimated the critical values of *CBF* that can lead to haemorrhage in the immature brain. This was done by analysing the biomechanical strength of brain capillaries and evaluating under which conditions of blood flow a rupture may occur. A single capillary was modelled as being detached from the surrounding connective tissue. Furthermore, it was specified that the capillaries have a cylindrical shape and walls composed of an endothelial and a basement layer. Linear elastic material properties were assumed for both layers, with elastic constants taken from the literature. The forces exerted on vessel walls—hydrostatic pressure and shear stress—were derived using the mathematical model^8,9,23^ based on the clinically measured values of *MAP* and *pCO*_*2*_. For each clinical measurement, the *FEM* was employed to calculate the von Mises stresses exerted on *GM* and non-*GM* capillaries. The critical *CBF* value leading to *IVH* was determined as the lower *CBF* value that maximized the performance of the *ROC*-based discrimination analysis between groups of preterm infants with and without *IVH*.

The maximum von Mises stress was observed in the basement membrane, as a result of the large difference between Young’s modules of the endothelial layer and basement membrane. This is in agreement with previous studies^15,16^ showing that the basement membrane is the main stabilizing and load bearing part of the capillary wall. The maximum von Mises stress, averaged across *WG*, was significantly higher in the *GM* capillaries than the non-*GM* capillaries. This result explains the more frequent origin of haemorrhages in the *GM*^2,3^.

No statistically significant difference in the von Mises stress was found between the control and affected groups, and therefore, the von Mises stress could not serve as a discrimination factor. For this reason, the discriminative *ROC* analysis in respect to *IVH* was performed using the percentage of damaged nodes ${N}^{F}\left({S}_{U}\right)$ of a *GM* capillary for ultimate stress ${S}_{U}$ in the range from 30 to 40 kPa. The statistical classification was effective for the very preterm infants with the gestational age of 27 to 30 *WG*, but not for extremely preterm infants with the gestational age of 23 to 26 *WG*. This result can be explained by the difference in the mean blood velocity, which was 2.3 times higher in the group of very preterm infants relative to extremely preterm infants. Blood velocity influences the shape and the motion of erythrocytes in narrow capillaries, with larger blood flow values inducing a more elongated shape of erythrocytes and a reduction in the diameter^27^. Thus, blood plasma flows around the deformed erythrocytes and creates a lubrication layer between the blood cells and the capillary wall. In this scenario, the rupture of a vessel wall is mainly caused by a high hydrostatic pressure during a period of arterial hypertension^6^. Therefore, the critical values of *CBF* underlying the potential occurrence of a disruption could be estimated by the *FEM* model developed.

For extremely preterm infants with the gestational age of 23 to 26 *WG*, it was not possible to determine a *CBF* critical value, likely due to the very low blood flow. At small velocities, erythrocytes do not smoothly flow through the capillary^27^, and their aggregation may rupture the vessel and produce a haemorrhage^28^. In this case, the *FEM* is not applicable, and the prediction of *IVH* has to be based on a critically low value of blood velocity.

For very preterm infants with the gestational age of 27 to 30 *WG*, the *CBF* = 17.03 ml/100 g/min was determined as the critical upper value. Thus, all the values of *MAP* and *pCO*_2_ for which the calculated *CBF* is greater than 17.03 ml/100 g/min indicate a higher likelihood of the development of *IVH*. Only slight variations of 5.7% of this critical value were obtained when either the boundary conditions employed in the model or the Young modulus of the basement membrane were changed. This suggests that the critical values obtained are stable for those alterations in the model. We can compare our results with animal studies qualitatively despite the fact that the absolute value of *CBF* varies among species^11^. Intraventricular haemorrhage has been attributed to changes in *CBF* leading to the damage of germinal matrix microvessels in preterm beagle puppies^12^. Also, impaired autoregulation and increased *CBF* have been associated with the risk of intraventricular haemorrhage in lambs and puppies^10,11,13^.

As mentioned above, a number of assumptions and simplifications were included in the *FEM* model employed. The geometry of individual capillaries was modelled as a perfectly uniform straight vessel without tissue support and with a continuous basement membrane. Although some studies have reported irregularities and discontinuities in the basement membrane^5,6^, the simplifications considered are unlikely to substantially change the results obtained, since the distinction between the control and affected groups was performed for the same gestational age and thus based on basement membranes with similar material properties. Furthermore, only two kinds of capillaries were modelled. No distinction was made between the periventricular and cortical capillary bed. Nevertheless, for the immature brain, where *GM* is still present, a detailed description of the different types of non-*GM* capillaries will not improve the results obtained because the stresses in the *GM* capillaries are significantly higher than those in other brain capillaries.

Another limitation of the model developed is the description of the material properties by a linear elastic model using material constants from the *AFM* experiments published. However, despite the fact that experimental conditions affect elastic properties and, therefore, the values of material constants, the discriminative analysis between the control and affected groups of preterm infants showed stable results for different values of the Young modulus. The development of nonlinear and visco-elastic models would nevertheless be a reasonable extension of the present study, although for fragile capillary vessels only small changes can be expected with such enhancements.

The biomechanical finite element modelling of stresses in cerebral capillaries combined with the mathematical model for computing *CBF* is a promising tool for the evaluation of the critical values of *CBF* and prediction of *IVH* in preterm infants. For very preterm infants with the gestational age of 27 to 30 weeks, all clinical values of *MAP* and *pCO*_2_ for which the calculated *CBF* is greater than 17.03 ml/100 g/min indicate a higher likelihood of the development of *IVH*. For extremely preterm infants with the gestational age of 23 to 26 weeks, the combination of methods developed was not able to determine a *CBF* critical value. It is likely due to the very low blood flow and, therefore, the prediction of *IVH* has to be based on a critically low value of blood velocity.

## Methods

The overall biomechanical behaviour of a blood vessel is determined by its structure, elastic properties of its constituent materials and by the forces that are exerted upon it. The following sections describe how the material properties and structure of vessels were mathematically modelled, and how the stress on vessel walls was computed. We start, however, by briefly describing the clinical data that allowed us to compute the hydrostatic pressure and the velocity of blood flow, both of which are then used to determine the stresses exerted on vessel walls.

### Description of clinical data

Medical data were obtained retrospectively from the standard clinical records of 254 preterm infants treated in the Departments of Neonatology of the University Hospital of the Technical University of Munich and of the University Hospital Essen of the University Duisburg-Essen. The study was approved by the Ethic Committees of the University Hospital of the Technical University of Munich (Ref. 364/15) and of the University Hospital Essen of the University Duisburg-Essen (Ref. 16-7284-BO). According to the rules of the Ethic Committees, no informed consent from parents was necessary for retrospective data from the existing medical files. All methods were performed in accordance with relevant guidelines and regulations.

The gestational age of the sample group ranged from 23 to 30 *WG* and the body weight from 335 to 1,580 g. In both clinical centres the occurrence of *IVH* was diagnosed using a standard transcranial ultrasound. Examinations were performed routinely on days 1, 3, 7, and 14 of life and more frequently (up to daily) in case of discrepancies or suspected haemorrhage. Patients without *IVH* (118) were here considered as control group and patients with *IVH* (136) as affected group (see Table 2). *MAP* and *pCO*_2_ were collected as standard medical measurements during routine clinical nursing for the first 10 days after birth in the control group, and for up to 7 consecutive days before and 3 days after haemorrhage in the affected group. Only arterial and capillary values of *pCO*_2_ were taken for the analysis. For each patient, *MAP* and *pCO*_2_ were measured at different time points and intervals. In numerical computations, however, only records taken during the same measurement procedure were used. The number of measurements per patient varied from 5 to 54 and the total number of coincident records of *MAP* and *pCO*_2_ was 3,240.

**Table 2:** Number of infants for different weeks of gestation, grades and the day of *IVH* diagnosis.

*WG*	*IVH*		*IVH* grade				Day of *IVH* diagnosis					
	No	With	I	II	III	IV	1st	2nd	3rd	4th	5th	> 5th
23	9	17	2	6	9	–	3	2	7	1	1	3
24	22	24	3	10	9	2	3	3	9	6	–	3
25	17	23	7	8	5	3	3	4	7	5	1	3
26	17	20	4	6	8	2	–	3	10	2	3	2
27	12	15	7	2	5	1	4	1	1	3	2	4
28	16	18	7	5	6	–	2	5	4	1	1	5
29	12	11	4	2	5	–	1	1	2	2	2	3
30	13	8	4	3	1	–	–	1	–	–	1	6
All	118	136	38	42	48	8	16	20	40	20	11	29

### Modelling of *CBF* in the immature brain

A mathematical model of *CBF*^8,9^ in the immature brain was derived from a hierarchical cerebrovascular model available for the adult brain^29^. In the model, the brain vascular system is divided in 19 hierarchical levels according to the morphological characteristics of the vessels. Different levels are connected in series and vessels in each level are represented as resistors, being connected in parallel within each level. The number of vessels as well as their lengths and diameters are scaled according to the brain weight of each infant, with this being estimated from the birth weight and gestation age. The presence of *GM* is modelled according to the gestational age as an additional parallel circuit at the capillary layer *n* = 10. The vascular response to a change in *MAP* and *pCO*_2_ is incorporated into the model through an increase/decrease in the diameter $d$ (i.e., vasodilation or vasoconstriction) of the vessel. The values of *CBF* estimated with the model developed, as well as its reaction to changes in *MAP* and *pCO*_2_, showed a good agreement with the equivalent experimental values taken from the literature^9^.

The model enables us to calculate the total *CBF* as follows:

$$CBF=CPP/\sum {R}_{n}.$$

The value of the *CBF* depends on the cerebral perfusion pressure (*CPP*), which is calculated as the difference between *MAP* and intracranial pressure ${P}_{ic}$^30,31^:

$$CPP=MAP-{P}_{ic}.$$

Unfortunately, intracranial pressure is not routinely monitored in neonates. For this reason, the constant value ${P}_{ic}$ = 1.7 kPa (17 cm H_2_O) taken from the literature^32^ was used in the numerical calculations for all infants.

The hydrostatic pressure $P$ on each level, *n* = 1…19, can be calculated^8^ as follows:

$${P}_{n+1}=MAP-CBF\cdot \sum {R}_{n}.$$

Here, ${P}_{1}=MAP$ and ${R}_{n}$ is the total vascular resistance of level *n* (*n* = 1…19).

The velocity $V$ of blood flow near the capillary wall can be evaluated as a function of the vessel’s radius $r$^23^ as:

$$V\left(r\right)=\Delta {P}_{10}\left({r}^{2}-{r}_{c}^{2}\right)/4{l}_{c}\mu,$$

and the resulting shear stress $\tau$ at the capillary wall can be calculated as follows:

$$\tau =\mu \cdot \partial V/\partial r=CBF\cdot {R}_{10}{r}_{c}/2{l}_{c}.$$

Here ${r}_{c}$ and ${l}_{c}$ are the radius and the length of the capillary, and μ = 0.001 Pa s is the dynamic blood viscosity near the capillary wall^23^.

### Material properties of capillary walls

Capillary walls have no muscle layer and their mechanical properties are determined by the microscopic structure of their constituent endothelial cells and basement membrane. In the present study, the description of biomechanical properties is based on *AFM* nanoindentation experiments on endothelial cells^14,20,21^ and the basement membrane^15,16,22^ found in the literature. The *AFM* allows the exploration of micro- and nanomechanical properties of cell structures. Living cells are rather soft and delicate, making investigation with *AFM* techniques under physiological conditions extremely challenging. The detailed schemes^14,20,21^ demonstrate a sequence of operations in the *AFM* probing that starts with the indentation of a sample, followed by recording the displacement, and ends with the force-curve analysis.

To indent the samples, the calibrated cantilever is used. In past experiments, different rates of indentation have been utilized. For instance, in the study of Ohashi et al.^14^, the force-indentation curve was obtained with the indentation rate of 880 nm/s. Marsh and Waugh^21^ repeated 20 indentations at a rate of 1 μm/s. In the study of Candiello et al.^15^, the speed of the *AFM* tip indenting the tissue was between 2.0 and 10.0 μm/s. The consecutive measurements were transformed into force-indentation curves that represent a basis for the estimation Young's elastic modulus. In order to determine this parameter, the force-indentation curves, which provide a relation between the loading force and the indentation, were fit with the Hertzian^14,16,21,22^, Sneddon^15,16^ or *FEM* models^14^.

*AFM* observations allow the possibility of studying the inner structure of vessel walls. Individual endothelial cells contain a cytoskeleton of actin stress fibres^18^ that can be identified as the load-bearing and force transmission element^33,34^. The topographically rich 3-dimensional paper-like network of fibres determines the local stiffness of the individual endothelial cells^14,33^, which are exposed to shear stresses resulting from blood flow^14,20,21^. The *AFM* experiments^14,20,21^ suggest a linear relationship between the deformations of endothelial surfaces and the amplitude of stress components. The measurement of the local compliance of endothelial cells demonstrated a significant increase of cells’ Young elastic modulus with shear stress^14,20,21^. The absolute values of elasticity parameters calculated using various models differed from each other, but the same tendency relative to exposure of shear stress was observed^20^. Young’s elastic modulus of the endothelium layer ${Y}_{E}$ was calculated in the present work as a function of shear stress $\tau$ as follows:

$${Y}_{E}\left(\tau \right)=-0.55{\tau }^{2}+4.35\tau +12.2.$$

The formula was obtained by fitting a polynomial to the experimental values taken from the literature: ${Y}_{E}$ = 12.2 kPa at $\tau$ = 0 (no shear force)^14^; ${Y}_{E}$ = 16 kPa at $\tau$ = 1 Pa^21^; and ${Y}_{E}$ = 18.7 kPa at $\tau$ = 2 Pa^14^.

Endothelial cells are surrounded by the basement membrane. Stresses are transmitted via the cytoskeleton from the endothelial layer to the underlying basement membrane, a felt-like meshwork containing pores and fibres arranged in an isotropic manner^19,34^. In the present work the basement membrane is described as an isotropic material with a Young’s modulus ${Y}_{M}$= 100 kPa, corresponding to the mean of the values available in the literature^15,16,19,22^. Both the endothelium and the basement membrane are furthermore almost incompressible materials. This property is expressed by the Poisson ratio *ν* that in case of the endothelium and basement membrane were given the following values: ${\nu }_{E}$ = 0.49^14^ and ${\nu }_{M}$ = 0.47, respectively^15^.

### Biomechanical forces acting on vessel walls

A single capillary is modelled here as a straight uniform cylinder, and blood flow is assumed to be constant, incompressible, and laminar. In this case, three forces are exerted on vessel walls: (1) the blood hydrostatic pressure $P$, (2) the shear stress $\tau$, and (3) the external interstitial fluid pressure ${P}_{EXT}$. The hydrostatic pressure $P$ acting on the inner surface of capillaries exerts a circumferential stress on the wall (hoop stress), and is perpendicular both to the axis and to the radius of the cylinder vessel. The shear stress $\tau$ depends on the velocity of blood flow and works as a tensile force in the longitudinal direction along the capillary wall. In the current study, the blood pressure $P$, the velocity of blood flow $V,$ and the shear stress $\tau$ in a single capillary were calculated from the clinically measured values of *MAP* and *pCO*_2_ with the hierarchical cerebrovascular model^8,9,23^, as described in “Modelling of *CBF* in the immature brain” section.

The external pressure ${P}_{EXT}$ corresponds to the pressure exerted by the interstitial fluid. In the present paper, no capillary exchange between blood and the interstitial fluid is considered. We assume, furthermore, that the interstitial hydrostatic pressure and blood colloid osmotic pressure are identical for all capillaries and remain steady. Hence, here the pressure acting on the external surface of capillaries was set to have a constant value of ${P}_{EXT}$= 6 mmHg (0.8 kPa)^25,26^. This value was selected from among those available in the literature, which differed depending on the method employed.

### Finite element model

The finite element method (*FEM*) was employed to calculate the stresses exerted on the capillary wall. With the *FEM* the different biomechanical properties of each layer of the vessel wall can be accounted for by assigning individual material properties to each finite element. Thus, the *FEM* may provide a more realistic simulation and description of the biomechanical conditions at the capillary level.

The *FEM* was implemented using the MATLAB R2019a library for structural analysis. The geometry of a single capillary was modelled as a two-layer cylinder. The diameter $d$ of the capillary was calculated by the mathematical model^8,9^ from the initial diameter ${d}_{0}$ using clinical record of *MAP* and *pCO*_*2*_. The initial diameter of *GM* capillaries was set to ${d}_{0}^{GM}$ = 6.8 μm and the length to ${l}^{GM}$ = 40 $\mu m$^5,7^, whilst for non-*GM* brain capillaries the initial values of diameter ${d}_{0}^{BR}$ = 5.6 $\mu m$ and of length ${l}^{BR}$ = 60 μm^29^ were used. For all capillaries, the thickness of the endothelium was set to ${t}_{E}$ = 0.4 μm, which is close to the measured value of 0.38 ± 0.05 μm^21^, and the thickness of the basement membrane to ${t}_{M}$ = 0.1 μm^16^.

The inner layer of the capillary was modelled with the material properties of endothelial cells taken from the literature^14,20,21^ (see “Material properties of capillary walls” section), with the Poisson ratio of ${\nu }_{E}$ = 0.49 and the shear stress-dependent Young modulus ${Y}_{E}\left(\tau \right)$. Since ${Y}_{E}\left(\tau \right)$ was determined from single-cell *AFM* measurements^14,20,21^ and the capillary endothelial layer has single-cell thickness, all mesh elements describing endothelial cells were given the same value of Young's modulus. This was calculated from the surface shear stress and remained constant throughout the computation. In other words, a zero approximation of Young’s modulus was used. The outer layer, in turn, incorporates the material properties of the basement membrane (see “Material properties of capillary walls” section), being characterized by the constant Young modulus ${Y}_{M}$ = 100 kPa and Poisson's ratio ${\nu }_{M}$ = 0.47.

A meshed geometry, with tetrahedral elements, was employed to describe the capillaries (Fig. 3a), and biomechanical stresses were calculated for each mesh node. The geometry, the loading forces (described in “Biomechanical forces acting on vessel walls” section), and, consequently, the stresses were assumed to be cylindrically symmetric and independent of the angular coordinate. Wall boundary conditions were furthermore set for capillaries, such that the radius of a vessel could change (i.e. vasodilation and/or vasoconstriction). Two kinds of boundary conditions on the length of the capillaries were employed: (1) one where the ends of the capillary were fixed at the junctions with other vessels on both sides; (2) and one where the capillary was fixed at one end and free at the other end.

### Analysis of critical parameters

When investigating mechanical failure of blood vessels, critical stresses are usually derived^35–37^ by comparing the von Mises stress ${S}_{VM}$ with the ultimate stress ${S}_{U}$ of the material, the maximum value of stress that a material can bear without failing. In the *FEM*, for each mesh node *i* (Fig. 3a), the von Mises stress ${S}_{VM}^{i}$ was defined using the computed principal stresses (*s*^*i*^_*1*_*, s*^*i*^_*2*_*, s*^*i*^_*3*_) as:

$${S}_{VM}^{i}=\sqrt{\left({\left({{s}^{i}}_{1}-{{s}^{i}}_{2}\right)}^{2}+{\left({{s}^{i}}_{2}-{{s}^{i}}_{3}\right)}^{2}+{\left({{s}^{i}}_{1}-{{s}^{i}}_{3}\right)}^{2}\right)/2},$$

Here it is assumed that a rupture in the mesh node *i* occurs if the condition ${S}_{VM}^{i}>{S}_{U}$ is satisfied. When the percentage of disrupted nodes ${N}^{F}$ exceeds the threshold value ${N}_{th}^{F}$, a global capillary damage leading to *IVH* is considered to occur. For each measurement, the percentage of the disrupted nodes ${N}^{F}$ of *GM* capillaries was calculated, and its maximum value for each patient was used as a classifier for discrimination between the control and affected groups using *ROC* analysis^38^. The *ROC* analysis evaluates the performance of a binary classifier based on the area under the curve (*AUC*: 1—perfect classifier, 0.7—good classifier, 0.5—random classifier^38^). The *ROC* curves were obtained by plotting the true positive rate (*TPR*, *Y* axis in Fig. 4b) versus the false positive rate (*FPR*, *X* axis in Fig. 4b) (definitions are provided in Table 3) for different threshold settings of ${N}_{th}^{F}$. The value of ${S}_{U}$ was a free parameter for *AUC* maximization. The optimal values of ${S}_{U}$ and ${N}_{th}^{F}$ minimising *AUC* were then used for the evaluation of the critical value of *CBF* leading to *IVH.* The critical *CBF* was set to be the lower limit across all *CBF* values that produced the optimal values of ${S}_{U}$ and ${N}_{th}^{F}$.

**Table 3:** Variables of *ROC* analyses.

Variable	Definition
*N*	The total number of measurements without *IVH*
*P*	The total number of measurements with *IVH*
*TP*	True positive (the number of correctly detected measurements with *IVH*)
*FP*	False positive (the number of measurements without *IVH* detected as with *IVH*)
*TPR* = *TP/P*	True positive rate
*FPR* = *FP/N*	False positive rate

Statistical results are presented as mean value plots and box plots with whiskers that are defined by the formulas: ${q}_{1}-1.5\cdot \left({q}_{3}-{q}_{1}\right)$, for the lower whiskers, and ${q}_{3}+1.5\cdot \left({q}_{3}-{q}_{1}\right)$, for the upper one, with *q*_*1*_ and *q*_*3*_ being the first and third quartiles, respectively. The *ROC* method as well as the Wilcoxon rank-sum test for statistical analyses were performed using standard functions of the statistical library of MATLAB R2019a.

### Testing effects of model assumptions

During the setup of the model several assumptions were done. The effect of these assumptions on the final results was verified by running the *FEM* for different conditions. Namely, given the lack of knowledge about the ability of capillaries to elongate longitudinally, both fixed and free boundary conditions for capillary ends were tested (see “Finite element model” section). Furthermore, due to the variety of Young's modulus values found in experimental literature for the basement membrane, the calculations were performed for two additional Young’s modulus values: for values 50% larger and smaller than the ${Y}_{M}$ value indicated in “Material properties of capillary walls” section.

### Ethics approval

The study was approved by the Ethic Committees of the University Hospital of the Technical University of Munich (Ref. 364/15) and of the University Hospital Essen of the University Duisburg-Essen (Ref. 16-7284-BO). All methods were performed in accordance with relevant guidelines and regulations.

### Consent for publication

According to the rules of the Ethic Committees of the University Hospital of the Technical University of Munich and of the University Hospital Essen of the University Duisburg-Essen, no informed consent from parents was necessary for retrospective data from the existing medical files.

## Data Availability

The dataset supporting the conclusions of this article is available in the mediaTUM, publications repository of the Technical University of Munich, https://mediatum.ub.tum.de/1521896.
